# Supplementary material for: Computational exploration of global venoms for antimicrobial discovery with Venomics artificial intelligence
Source: Nat Commun. 2025 Jul 12;16:6446. doi: 10.1038/s41467-025-60051-6 (PMC12254355; doi:10.1038/s41467-025-60051-6)
Supplement: Supplementary file 1 — Supplementary Information [file 41467_2025_60051_MOESM1_ESM.pdf]

Supplementary Information

Computational exploration of global venoms for antimicrobial discovery with Venomics artificial intelligence

Changge Guan †, Marcelo D. T. Torres†, Sufen Li, and Cesar de la Fuente-Nunez*

Corresponding autor: Cesar de la Fuente-Nunez (cfuente@upenn.edu)

Supplementary Information:
Supplementary Tables 1-4
Supplementary Figures 1-8

Supplementary Table 1. Database-sourced venom protein and VEP candidates.

**Database**	**Number of proteins mined**	**Number of candidate antimicrobials (MIC ≤32 μmol L^{-1})**	**Number of candidates removed by similarity to known AMPs**	**Candidates (diversity filter)**
ConoServer	5494	377	377	26
ArachnoServer	2206	2205	2154	80
ISOB	654	179	40	7
VenomZone (UniProtPK)	7769	4618	3731	273

Supplementary Table 2. Antimicrobials selected for synthesis and experimental validation.

Peptide	Sequence	Peptide	Sequence
UniprotKB-1	KLLKIGLKSFARVLKKVL	Conoserver-3	RRASPLWKRRRFLSMLKARAKRTGYK
UniprotKB-2	WLGSALKIGAKLL	Conoserver-4	PLWKRRRFLSMLKARAKR
UniprotKB-3	KLWNSKLARKIRTKGLKYVKNFAK	Conoserver-5	LRAKMLNSKFIKL
UniprotKB-4	LKLKSILGKLGVIL	Conoserver-6	KLHGLLTRRSLKNFWKRNLYLR
UniprotKB-5	RRVKRFKKFFMKLKKSVKKRVMKFFK	Conoserver-7	KRGRASPLWQRRGFLSKLKARAKRNGAFHLPR
UniprotKB-6	GKWLISSLVAKHL	Conoserver-10	RLRAKMRNSKLFKLTKR
UniprotKB-7	KRLKGFAKKLWNSKLARKIRTKGLKYVKNFAK	Conoserver-12	RQEYPTKRLRAKMLNSKFIKLIKR
UniprotKB-8	KLKKLRKWIYRIV	Conoserver-14	KKWRELSRLSRVLQIL
UniprotKB-9	FLKKIWRSKLVKRL	Conoserver-15	RKRRRFISMLKARAKRR
UniprotKB-10	KRRRASPLWKRRRFLSMLKARAK	Conoserver-16	KQKYLIKRSRAKMQNHKLFKLTKR
UniprotKB-11	LTKWLGKLGVIL	Arachnoserver-1	KLLKIGLKSFARVLKKVL
UniprotKB-12	RKFKWGKLFSTAKKLYKKGKKLSKNKNFKKALK	Arachnoserver-2	RKFKWGSFKKILSAGKKLFKKAKKLSK
UniprotKB-13	KFLARLVFRKFILL	Arachnoserver-4	KWGKLFSAGKKLLKKAKKL
UniprotKB-14	KNKRFIRNLRSNLYQKIIKSTKSLL	Arachnoserver-5	KIKWLKAMKSIAKFIAKK
UniprotKB-15	KWLGKLGVILSHL	Arachnoserver-6	RKFKWGSFKKILSAGKKLFKKAKKLSKNKNFKKALK
UniprotKB-16	RKFKWGKLFSTAKKLYKKGKKLSK	Arachnoserver-7	RKFNWGKLFKSAKKLYKTGKKLSKNKNVRKALKFGK
UniprotKB-17	FIKKLWRSKLAKKLRAKGRELLK	Arachnoserver-9	KNKRFIRNLRSNLYQKIIKSTKSLL
UniprotKB-18	RRVKRFKKFFMKL	Arachnoserver-11	ARKFKWGKLFSAGKKLLKKAKKLSKNK
UniprotKB-19	VNSFKIGGFIKKLWRSKLAKKLRAK	Arachnoserver-12	RGLAKLLKIGLKSFARVLKK
UniprotKB-20	RFGSFLKKVWKSKLAKKL	Arachnoserver-17	KRFIRNLRSNLYQKIIKSTKSLLDLREKI
ISOB-1	RRVKRFKKFFRKLKKSVKKRAKEFFK	Arachnoserver-18	KFSVFSKILRSIAKVF
ISOB-2	RHRIVRTYIAKFGLK	Arachnoserver-19	SKKQIRLYLLKYYGKKLFKKRPK
ISOB-3	KRKGYLRLVPEERIWQKGLWWLRRLETDSDKLQK	Arachnoserver-20	KKQIRLYLLKYYGKKSSSKSVRKIVISK
ISOB-4	LLHFSIWRSTVLRK	Arachnoserver-27	KFSVFSKILRSIAKVFKGVGKVRK
ISOB-5	RHRIVRTYIAKFGLKLNEFFQENENAWYFIRNIRKRVWEVKK	Arachnoserver-28	KLSGISKVLRAIAKFFK
ISOB-6	RRVKRFKKFFKKL	Arachnoserver-29	SFKKILSAGKKLFKKAKKL
ISOB-7	QPRRVKRFKKFFKKLKNSVKKRAKKF	Arachnoserver-31	KYRRGVSPWLKKELVRLHNNLRSKVAGGK
Conoserver-1	KRLRAKMLNSKFIKLIKR	Arachnoserver-32	KWLKAMKSIAKFIAKKQMKKHL
Conoserver-2	KRRRASPLWKRRRFLSMLKARAK	Arachnoserver-33	KIKWFKTMKSLAKFLAK

Supplementary Table 3. Selectivity index (SI) of VEPs. SI was calculated based on the cytotoxicity against HEK293Tcells (CC_{50}), red blood cells (HC_{50}), and lowest MIC value. All values are presented as μmol L^{-1}.

Peptide	MIC	CC_{50}	$SI_{cytotoxicity}$	HC_{50}	$SI_{hemolysis}$	Peptide	MIC	CC_{50}	$SI_{cytotoxicity}$	HC_{50}	$SI_{hemolysis}$
UniprotKB-1	4	10.1	2.5	34.9	8.7	Conoserver-3	32	193.2	6.0	459.2	14.4
UniprotKB-2	-	8.3	-	5.529e+042	-	Conoserver-4	32	83.51	2.6	707.7	22.1
UniprotKB-3	8	11.6	1.5	8.23e+034	1.02875E+34	Conoserver-5	-	156.1	-	1.085e+083	-
UniprotKB-4	16	10.5	0.7	3.938e+054	2.46125E+53	Conoserver-6	4	31.7	7.9	66.3	16.6
UniprotKB-5	8	8.9	1.1	103.8	13.0	Conoserver-7	32	79.8	2.5	1.085e+083	3.39063E+81
UniprotKB-6	64	19.5	0.3	686.0	10.7	Conoserver-10	-	76.2	-	1.085e+083	-
UniprotKB-7	4	14.8	3.7	207.9	52.0	Conoserver-12	16	93.6	5.8	1.085e+083	6.78125E+81
UniprotKB-8	8	27.6	3.5	636.4	79.6	Conoserver-14	8	58.5	7.3	1826.0	228.3
UniprotKB-9	16	20.2	1.3	219457.0	13716.1	Conoserver-15	32	22.5	0.7	1.085e+083	3.39063E+81
UniprotKB-10	32	43.1	1.3	627.2	19.6	Conoserver-16	64	93.2	1.5	1.085e+083	1.69531E+81
UniprotKB-11	32	140.4	4.4	146.7	4.6	Arachnoserver-1	4	26.2	6.6	82.7	20.7
UniprotKB-12	2	23.7	11.9	604.3	302.2	Arachnoserver-2	1	9.9	9.9	108.1	108.1
UniprotKB-13	2	26.7	13.4	455.9	228.0	Arachnoserver-4	8	19.6	2.4	1.085e+083	1.35625E+82
UniprotKB-14	8	45.9	5.7	7.733e+045	9.66625E+44	Arachnoserver-5	2	14.0	7.0	46.7	23.3
UniprotKB-15	32	46.0	1.4	1.374e+045	4.29375E+43	Arachnoserver-6	4	5.2	1.3	12.1	3.0
UniprotKB-16	4	23.7	5.9	2082.0	520.5	Arachnoserver-7	1	6.8	6.8	7.0	7.0
UniprotKB-17	8	31.0	3.9	4.224e+042	5.28E+41	Arachnoserver-9	8	23.6	2.9	4947.0	618.4
UniprotKB-18	32	99.7	3.1	1.827e+053	5.70938E+51	Arachnoserver-11	4	8.7	2.2	1118.0	279.5
UniprotKB-19	4	40.6	10.2	625.9	156.5	Arachnoserver-12	4	8.4	2.1	40.1	10.0
UniprotKB-20	8	52.5	6.6	18147.0	2268.4	Arachnoserver-17	4	16.5	4.1	280.5	70.1
ISOB-1	4	115.6	28.9	2001.0	500.3	Arachnoserver-18	4	12.3	3.1	53.5	13.4
ISOB-2	32	241.3	7.5	1.085e+083	3.39063E+81	Arachnoserver-19	8	16.7	2.1	163.3	20.4
ISOB-3	-	120.7	-	1.085e+083	-	Arachnoserver-20	32	91.1	2.8	896.7	28.0
ISOB-4	16	154.6	9.7	24308.0	1519.3	Arachnoserver-27	4	8.0	2.0	24.2	6.0
ISOB-5	-	1.338E+43	-	2549.0	-	Arachnoserver-28	8	6.3	0.8	38.2	4.8
ISOB-6	32	209.5	6.5	1.085e+083	3.39063E+81	Arachnoserver-29	4	17.1	4.3	1.085e+083	2.7125E+82
ISOB-7	32	129.0	4.0	569.0	17.8	Arachnoserver-31	4	12.7	3.2	1392.0	348.0
Conoserver-1	16	82.56	5.2	1.085e+083	6.78125E+81	Arachnoserver-32	4	7.6	1.9	19.2	4.8
Conoserver-2	32	115.0	3.6	1570.0	49.1	Arachnoserver-33	4	32.1	8.0	144.1	36.3

Supplementary Table 4. Prediction of experimental verification VEPs modulating ion channel.

Peptide	Pred_tgt _calcium	Pred_tgt_ nAChRs	Pred_tgt_ potassium	Pred_tgt_ sodium	Peptide	Pred_tgt_ calcium	Pred_tgt_ nAChRs	Pred_tgt_ potassium	Pred_tgt_ sodium
UniprotKB-1	-	-	Modulate	-	Conoserver-3	-	-	-	-
UniprotKB-2	-	-	Modulate	-	Conoserver-4	-	-	Modulate	-
UniprotKB-3	-	-	-	-	Conoserver-5	-	-	Modulate	-
UniprotKB-4	-	-	Modulate	-	Conoserver-6	-	-	Modulate	-
UniprotKB-5	-	-	Modulate	-	Conoserver-7	-	-	-	-
UniprotKB-6	-	-	Modulate	-	Conoserver-10	-	-	Modulate	-
UniprotKB-7	-	-	-	-	Conoserver-12	-	-	-	-
UniprotKB-8	-	-	Modulate	-	Conoserver-14	-	-	Modulate	-
UniprotKB-9	-	-	Modulate	-	Conoserver-15	-	-	Modulate	-
UniprotKB-10	-	-	Modulate	-	Conoserver-16	-	-	-	-
UniprotKB-11	-	-	Modulate	-	Arachnoserver-1	-	-	Modulate	-
UniprotKB-12	-	-	-	-	Arachnoserver-2	-	-	-	-
UniprotKB-13	-	-	Modulate	-	Arachnoserver-4	-	-	Modulate	-
UniprotKB-14	-	-	-	-	Arachnoserver-5	-	-	Modulate	-
UniprotKB-15	-	-	Modulate	-	Arachnoserver-6	-	-	-	-
UniprotKB-16	-	-	Modulate	-	Arachnoserver-7	-	-	-	-
UniprotKB-17	-	-	-	-	Arachnoserver-9	-	-	-	-
UniprotKB-18	-	-	Modulate	-	Arachnoserver-11	-	-	-	-
UniprotKB-19	-	-	-	-	Arachnoserver-12	-	-	Modulate	-
UniprotKB-20	-	-	Modulate	-	Arachnoserver-17	-	-	-	-
ISOB-1	-	-	-	-	Arachnoserver-18	-	-	Modulate	-
ISOB-2	-	-	Modulate	-	Arachnoserver-19	-	-	Modulate	-
ISOB-3	-	-	-	-	Arachnoserver-20	-	-	-	-
ISOB-4	-	-	Modulate	-	Arachnoserver-27	-	-	Modulate	-
ISOB-5	-	-	-	-	Arachnoserver-28	-	-	Modulate	-
ISOB-6	-	-	Modulate	-	Arachnoserver-29	-	-	Modulate	-
ISOB-7	-	-	Modulate	-	Arachnoserver-31	-	-	-	-
Conoserver-1	-	-	Modulate	-	Arachnoserver-32	-	-	-	-
Conoserver-2	-	-	Modulate	-	Arachnoserver-33	-	-	Modulate	-

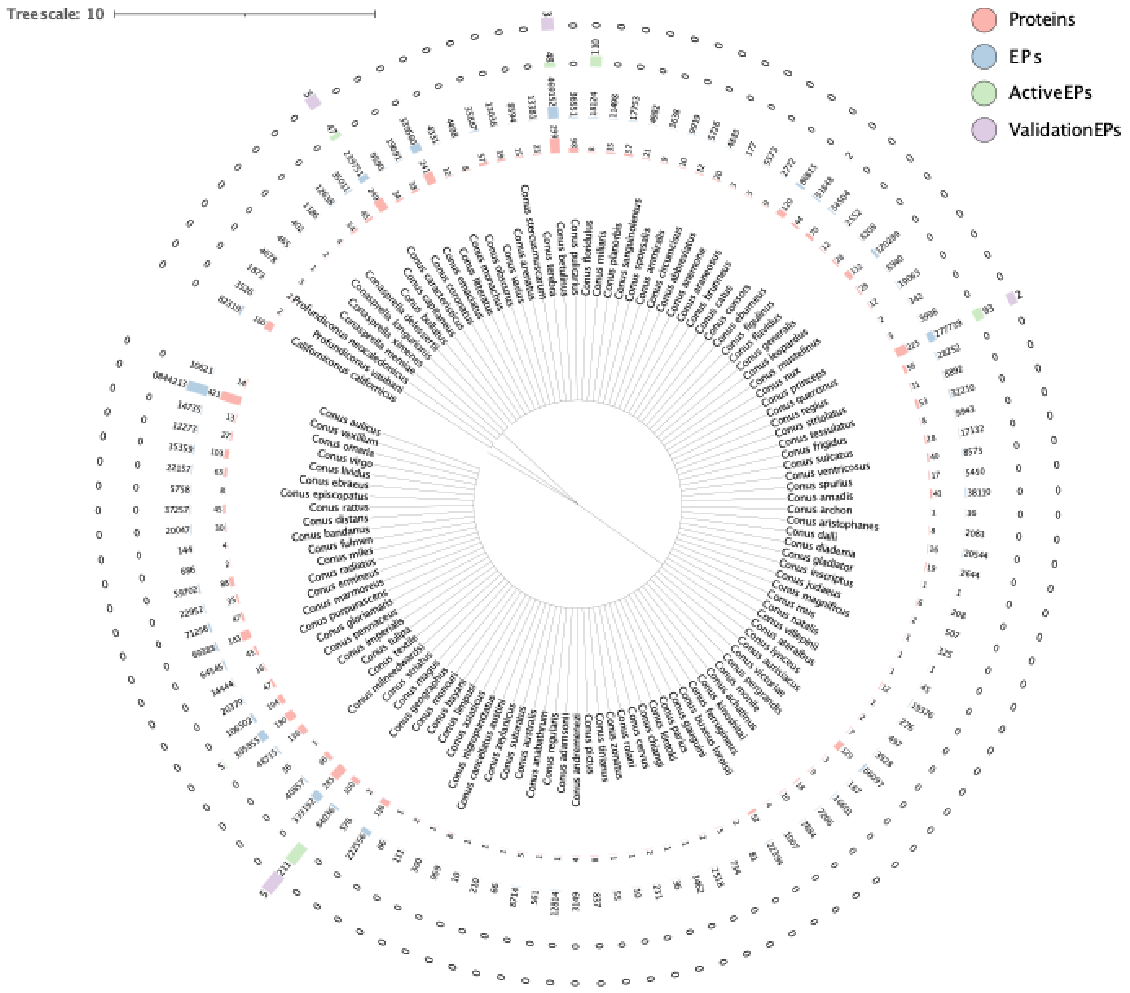

Supplementary Figure 1. Phylogenetic analysis of venom protein, peptide, predicted AMP, and verified AMP across species in ConoServer. This phylogenetic tree illustrates the distribution of venom proteins, peptides, predicted antimicrobial peptides (AMPs), and experimentally verified AMPs among species in ConoServer. The tree was constructed using taxon IDs of organisms. From the inside to the outside, circle 1: Venom protein count per organism; Circle 2: Peptide count derived from venom proteins per organism; Circle 3: Predicted AMP count from venom proteins per organism; Circle 4: Experimentally verified AMP count per organism.

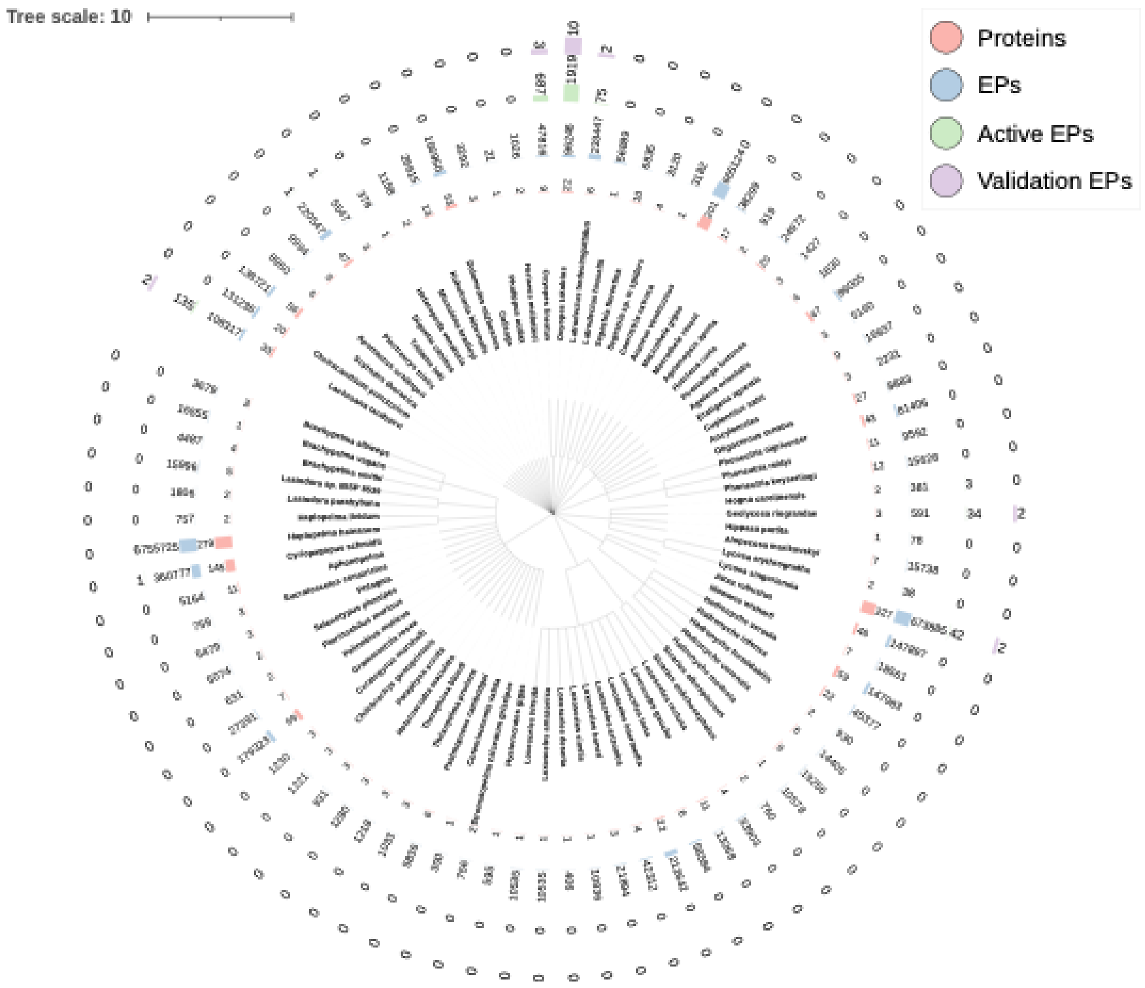

Supplementary Figure 2. Distribution analysis of venom protein, peptide, predicted AMP, and verified AMP across species in ArachnoServer. This phylogenetic tree illustrates relationship distance of different organisms. The tree was constructed using taxon IDs of organisms. From the inside to the outside, circle 1: Venom protein number; Circle 2: Peptide number; Circle 3: Predicted AMP number; Circle 4: Experimentally verified AMP number.

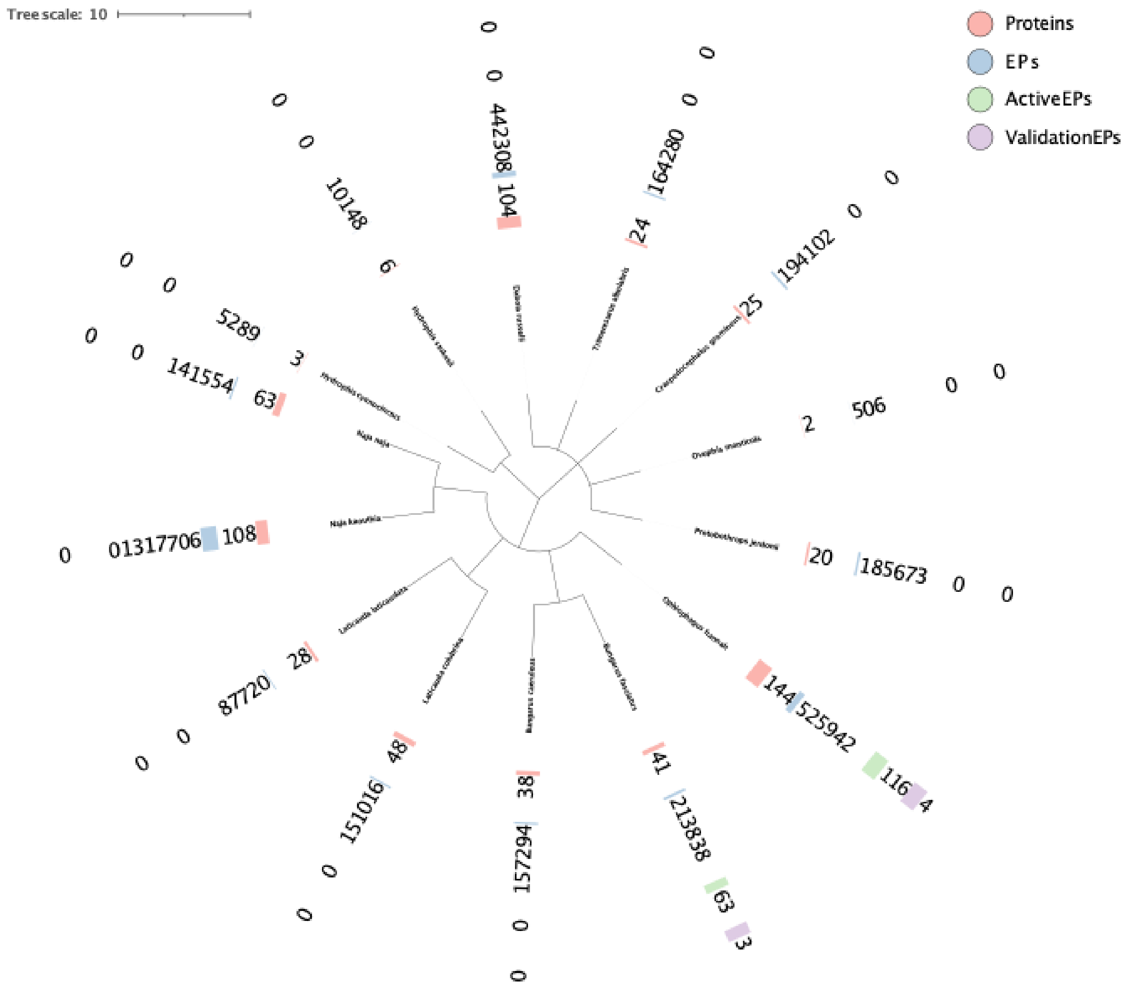

Supplementary Figure 3. The distribution of venom protein, peptide, predicted AMP, and verified AMP across species in ISOB. This phylogenetic tree illustrates the phylogenetic relationship of species in ConoServer, which contained the number of venom proteins, peptides, predicted antimicrobial peptides (AMPs), and experimentally verified AMPs among. The four circles represent, from the inside to the outside, number of venom protein, number of peptides, number of predicted AMP, number of experimentally validation AMP.

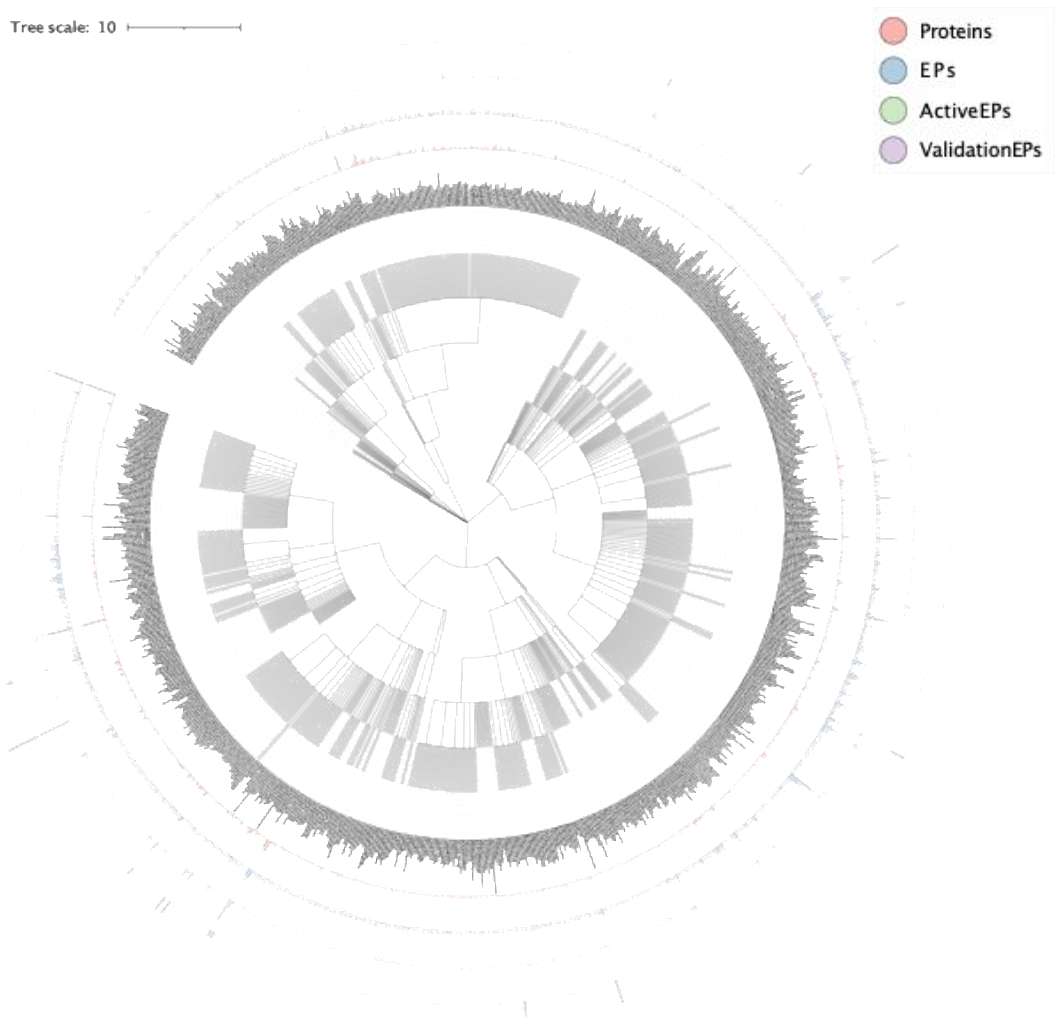

Supplementary Figure 4. Phylogenetic analysis of species in UniProt and distribution analysis of the number of venom protein, peptide, predicted AMP, and verified AMP across species in UniProt. This evolutionary tree shows the evolutionary relationships between species in ConoServer. The four circles represent, from the inside to the outside, the number of venom proteins contained in each organism, the number of peptides produced by the venom proteins of each organism, the number of predicted AMPs contained in each organism, and the number of experimentally verified AMPs contained in each organism.

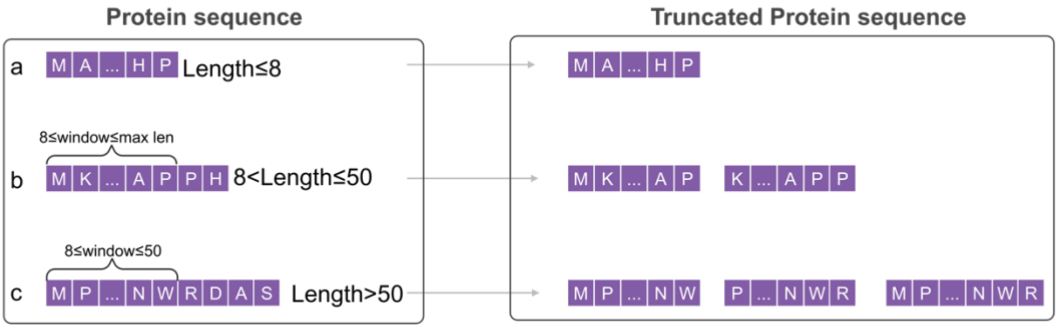

Supplementary Figure 5. Sequence truncation method. (a) Sequences with a length of 8 or fewer residues were retained without truncation. **(b)** Sequences with a length greater than 8 but not exceeding 50 were segmented using a sliding window approach to generate fragments ranging in size from 8 residues up to the full sequence length. **(c)** Sequences with a length of 50 or more were truncated using a sliding window approach to generate fragments with sizes ranging from 8 to 50 residues.

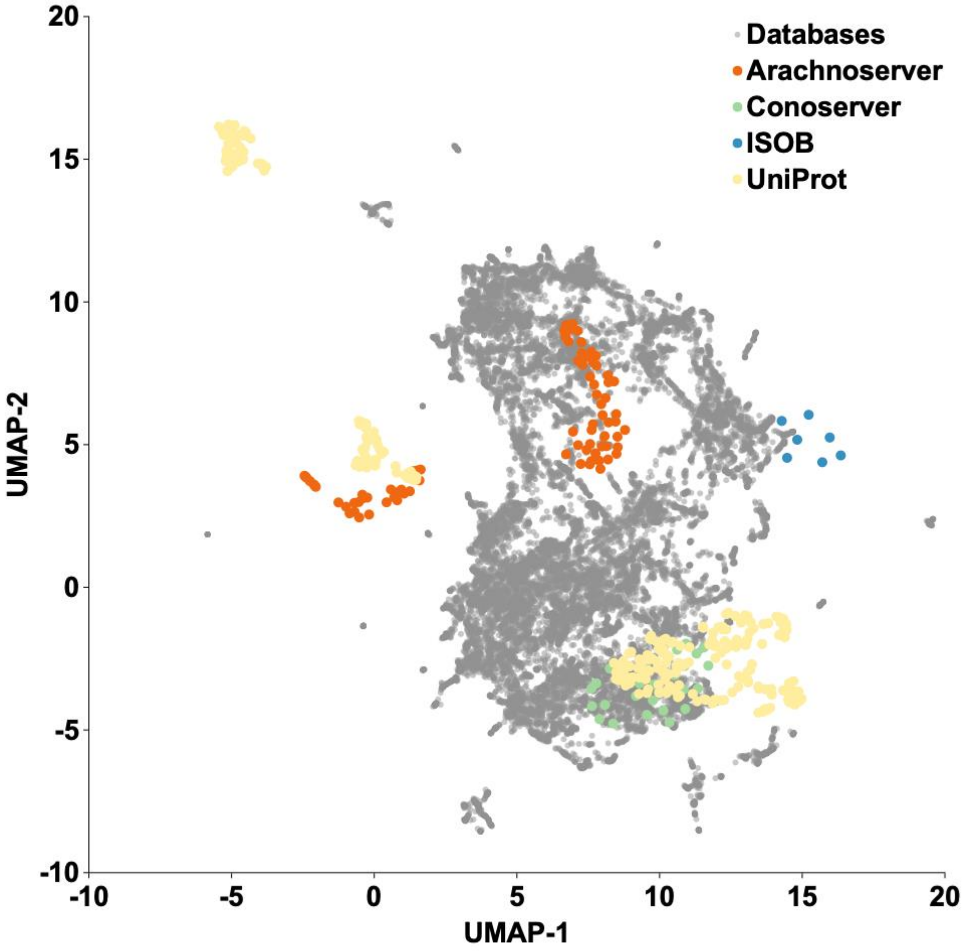

Supplementary Figure 6. Physicochemical properties space exploration using a similarity matrix. The graph illustrates a bidimensional physicochemical properties space visualization of peptide sequences found in DBAASP and antimicrobial venom-derived EPs (VEPs) discovered by APEX in venom proteins from multiple source organisms. Sequence alignment was used to generate a similarity matrix for all peptide sequences in DBAASP and the predicted antimicrobial VEPs (see also **Data S1**). Each row in the matrix represents a feature representation of a peptide based on its amino acid composition. Uniform Manifold Approximation and Projection (UMAP) was applied to reduce the feature representation to two dimensions for visualization.

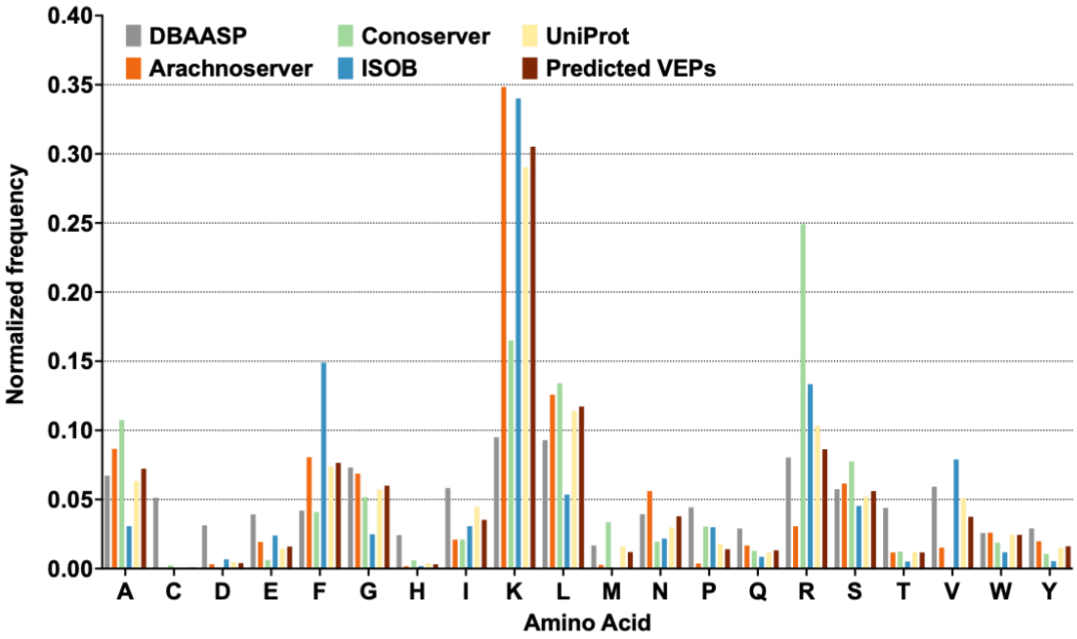

Supplementary Figure 7. VEPs amino acid frequency at amino acid residues level. Comparison between VEPs and known antimicrobial peptides (AMPs) from the DBAASP, APD3, and DRAMP 3.0 databases at amino acid level.

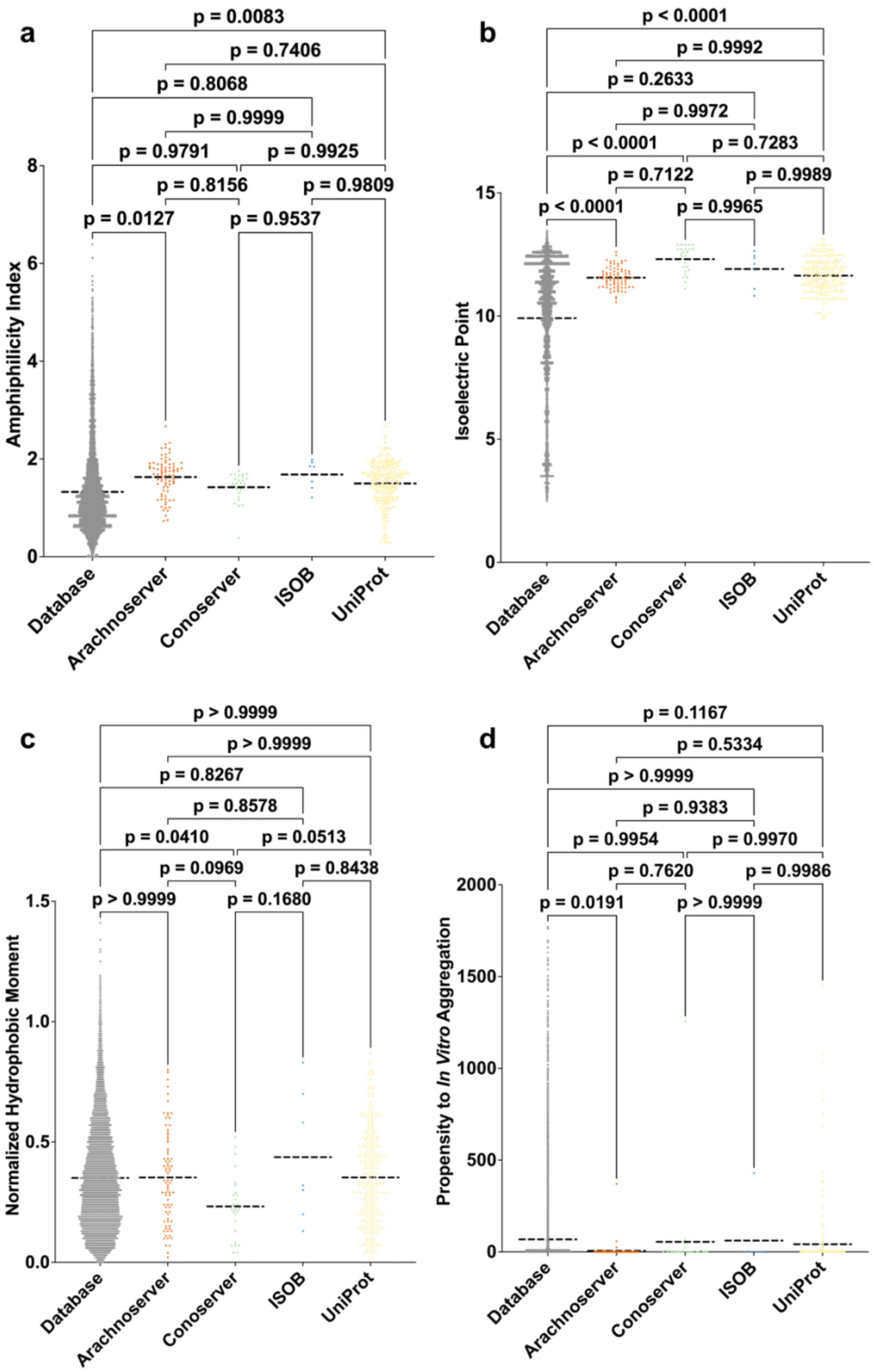

Supplementary Figure 8. Physicochemical features of VEPs compared to AMPs from databases (DBAASP, APD3, and DRAMP 3.0). (a) Amphiphilicity Index, **(b)** Isoelectric Point, and **(c)** Hydrophobic moment normalized by peptide length, reflecting the amphipathicity of the molecules, which directly influences their interactions with

bacterial membranes. **(d)** Propensity to aggregate *in vitro*, correlated with the supramolecular arrangement of the molecules and potential toxicity. Statistical significance was determined using two-tailed t-tests followed by the Mann-Whitney test; p values are shown in the graph. The solid line within each box represents the mean value for each group.

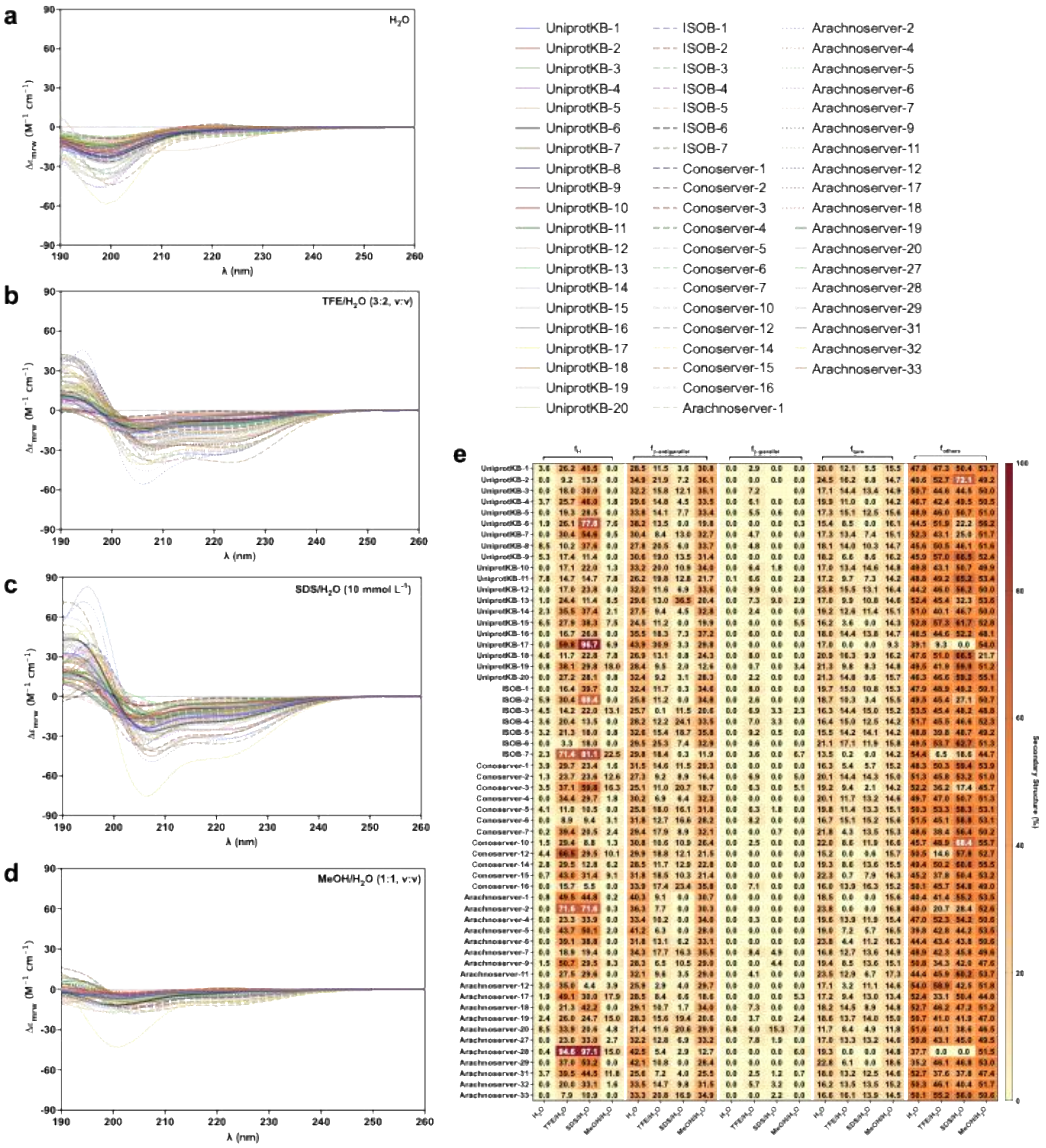

Supplementary Figure 9. Circular dichroism spectra of VEPs. Circular dichroism experiments were conducted with peptides from venoms using a J-1500 Jasco circular dichroism spectrophotometer. The spectra were recorded in four different media: **(a)** water, **(b)** 60% trifluoroethanol in water, and **(c)** sodium dodecyl sulfate (SDS) in water (10 mmol L^{-1}), and **(d)** 50% methanol in water, after three accumulations at 25 ^{o}C, using a 1mm path length quartz cell, between 260 and 190 nm at 50 nm min^{-1}, with a bandwidth of 0.5 nm. The concentration of all peptides tested was 50 µmol L^{-1}. **(e)** Heatmap with the percentage of secondary structure found for each peptide in the four different solvents. Secondary structure fraction was calculated using the BeStSel server[39].

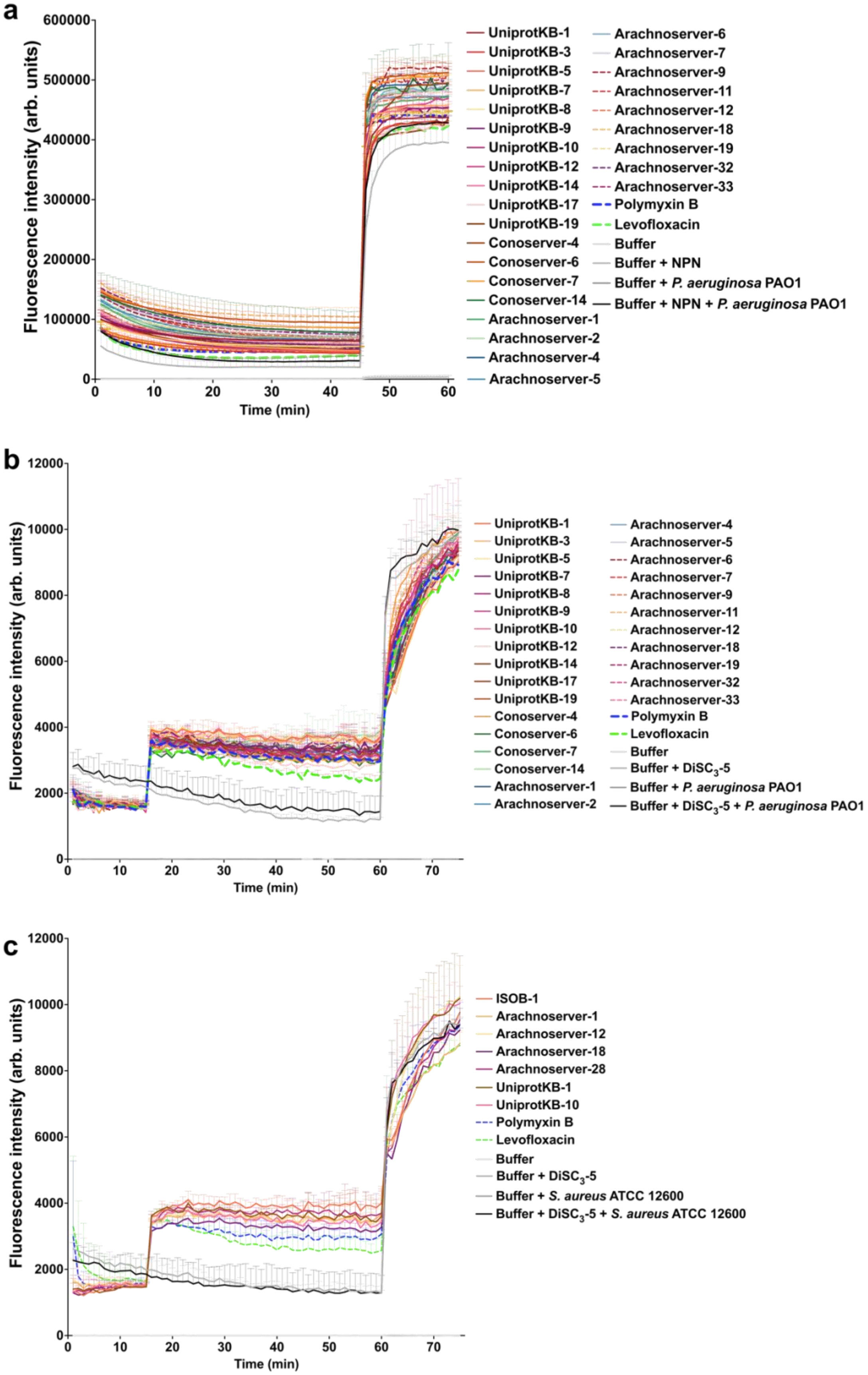
a
Fluorescence intensity (arb. units)
Time (min)
UniprotKB-1
UniprotKB-3
UniprotKB-5
UniprotKB-7
UniprotKB-8
UniprotKB-9
UniprotKB-10
UniprotKB-12
UniprotKB-14
UniprotKB-17
UniprotKB-19
Conoserver-4
Conoserver-6
Conoserver-7
Conoserver-14
Arachnoserver-1
Arachnoserver-2
Arachnoserver-4
Arachnoserver-5
Arachnoserver-6
Arachnoserver-7
Arachnoserver-9
Arachnoserver-11
Arachnoserver-12
Arachnoserver-18
Arachnoserver-19
Arachnoserver-32
Arachnoserver-33
Polymyxin B
Levofloxacin
Buffer
Buffer + NPN
Buffer + P. aeruginosa PAO1
Buffer + NPN + P. aeruginosa PAO1
b
Fluorescence intensity (arb. units)
Time (min)
UniprotKB-1
UniprotKB-3
UniprotKB-5
UniprotKB-7
UniprotKB-8
UniprotKB-9
UniprotKB-10
UniprotKB-12
UniprotKB-14
UniprotKB-17
UniprotKB-19
Conoserver-4
Conoserver-6
Conoserver-7
Conoserver-14
Arachnoserver-1
Arachnoserver-2
Arachnoserver-4
Arachnoserver-5
Arachnoserver-6
Arachnoserver-7
Arachnoserver-9
Arachnoserver-11
Arachnoserver-12
Arachnoserver-18
Arachnoserver-19
Arachnoserver-32
Arachnoserver-33
Polymyxin B
Levofloxacin
Buffer
Buffer + DiSC3-5
Buffer + P. aeruginosa PAO1
Buffer + DiSC3-5 + P. aeruginosa PAO1
c
Fluorescence intensity (arb. units)
Time (min)
ISOB-1
Arachnoserver-1
Arachnoserver-12
Arachnoserver-18
Arachnoserver-28
UniprotKB-1
UniprotKB-10
Polymyxin B
Levofloxacin
Buffer
Buffer + DiSC3-5
Buffer + S. aureus ATCC 12600
Buffer + DiSC3-5 + S. aureus ATCC 12600

Supplementary Figure 10. Outer membrane permeabilization and cytoplasmic membrane depolarization of *P.aeruginosa* PAO1 and *S. aureus* ATCC 12600 induced by VEPs. (a) Outer membrane permeabilization was assessed using the probe 1-(N-phenylamino)naphthalene (NPN), showing the permeabilization effects of VEPs active against *P. aeruginosa* PAO1. **(b-c)** Membrane depolarization assays were performed using the hydrophobic probe 3,3′-dipropylthiadicarbocyanine iodide ($DiSC_3$-5) on all VEPs active against **(b)** *P. aeruginosa* PAO1 and **(c)** *S. aureus* ATCC 12600. Polymyxin B and levofloxacin served as antibiotic controls, while buffer, buffer with the probe, and buffer with both probe and bacteria were used as baseline controls for fluorescence. The panels display the raw fluorescence intensity data obtained from the experiments. Error bars are the standard deviation obtained from the three replicates.

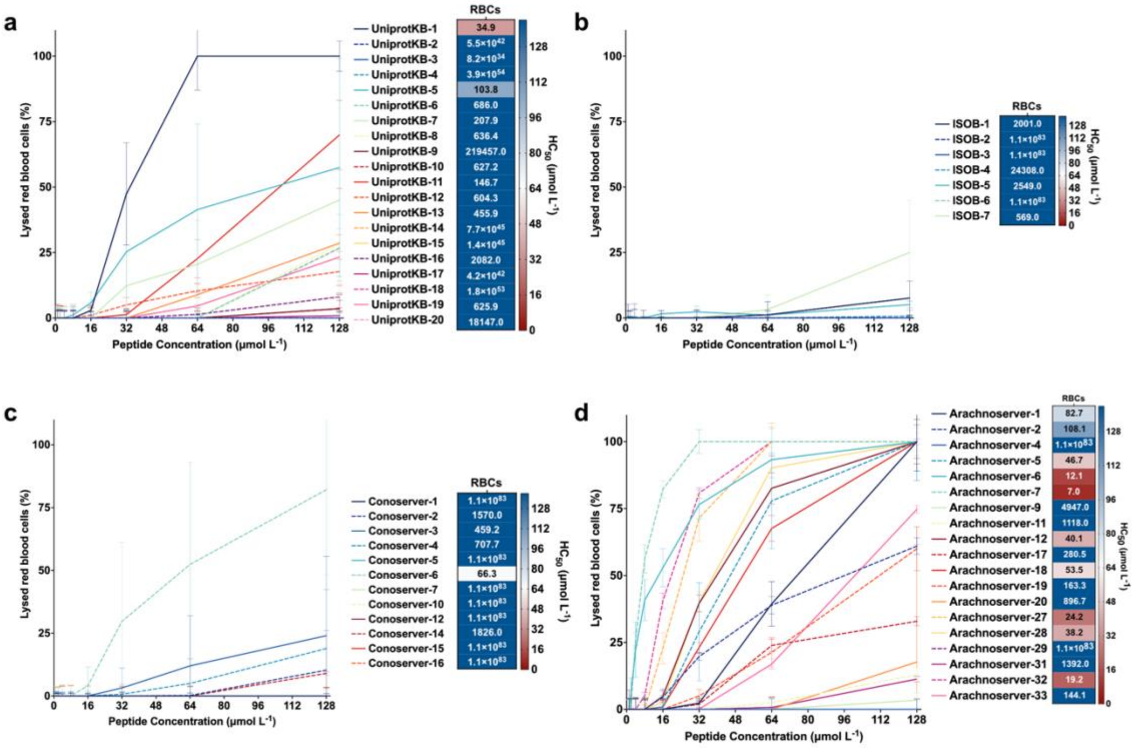

Supplementary Figure 11. Hemolytic activity induced by VEPs in human red blood cells. VEPs from **(a)** UniportKB, **(b)** ISOB, **(c)** Conoserver, and **(d)** Arachnoserver were exposed to human red blood cells (RBCs). Graphs show the percentage of lyzed RBCs after exposure. HC_{50} values were derived from dose-response curves obtained via non-linear regression analysis, representing the concentrations required to kill 50% of the cells in the experiment. Error bars are the standard deviation obtained from the three replicates.

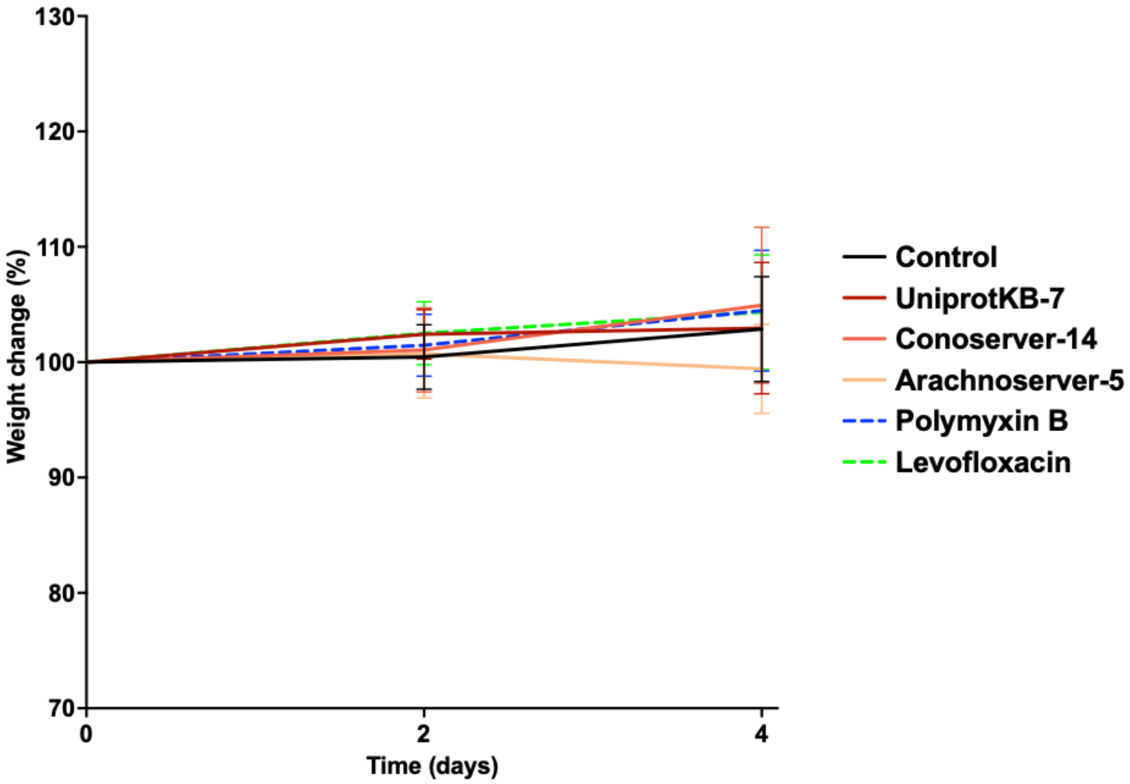

Supplementary Figure 12. Weight change monitoring in skin abscess mouse model infected with *A. baumannii*. Mouse weight was monitored throughout the duration of the skin abscess model (4 days total) to assess potential toxic effects of both the bacterial load and the VEPs.
