# Supplementary material for: Computational exploration of global venoms for antimicrobial discovery with Venomics artificial intelligence
Source: Nat Commun. 2025 Jul 12;16:6446. doi: 10.1038/s41467-025-60051-6 (PMC12254355; doi:10.1038/s41467-025-60051-6)
Supplement: Supplementary file 2 — Description Of Additional Supplementary File [file 41467_2025_60051_MOESM2_ESM.pdf]

Description of Additional supplementary files

Supplementary data 1:

List of VEP predicted by APEX to have a median MIC ≤32 µmol⁻¹.csv

Supplementary data 2:

List of VEP identified by APEX and filter criterion
